# Removal of urothelium affects bladder contractility and release of ATP but not release of NO in rat urinary bladder

**DOI:** 10.1186/1471-2490-10-10

**Published:** 2010-05-24

**Authors:** Alvaro Munoz, David A Gangitano, Christopher P Smith, Timothy B Boone, George T Somogyi

**Affiliations:** 1 Scott Department of Urology, Baylor College of Medicine, One Baylor Plaza, Houston, Texas, USA; 2 Department of Urology, The Methodist Hospital, 6560 Fannin Street, Houston, Texas, USA

## Abstract

**Background:**

The objective of our work was to investigate both the contractile function and the release of ATP and NO from strips of bladder tissue after removal of the urothelium.

**Methods:**

The method of removal was a gentle swabbing motion rather than a sharp surgical cutting to separate the urothelium from the smooth muscle. The contractile response and ATP and NO release were measured in intact as well as on swabbed preparations. The removal of the urothelial layer was affirmed microscopically.

**Results:**

After the swabbing, the smaller contractions were evoked by electrical as well as by chemical stimulation (50 μM carbachol or 50 μM α, β meATP). Electrical stimulation, carbachol and substance P (5 μM) evoked lower release of ATP in the swabbed strips than in intact strips. Although release of NO evoked by electrical stimulation or substance P was not changed, release of NO evoked by carbachol was significantly less in the swabbed preparations.

**Conclusion:**

Since swabbing removes only the urothelium, the presence of the suburothelial layer may explain the difference between our findings and those of others who found an increase in contractility. Evoked release of ATP is reduced in swabbed strips, indicating that ATP derives solely from the urothelium. On the other hand, electrical stimulation and substance P evoke identical degrees of NO release in both intact and swabbed preparations, suggesting that NO can be released from the suburothelium. Conversely, carbachol-induced release of NO is lower in swabbed strips, implying that the cholinergic receptors (muscarinic or nicotinic) are located in the upper layer of the urothelium.

## Background

The bladder urothelium is equipped with receptors for various neurotransmitters such as muscarinic, nicotinic [1], beta adrenergic [2], vanilloid [3], and purinergic receptors [4]. These receptors can be activated by their corresponding agonists to evoke substantial changes in bladder sensory mechanisms [1,5]. ATP and NO have also been shown to be among the major neurotransmitters that can be released from the urothelium following chemical [2,3,6] or osmotic stimulation [2,7].

ATP can be released from the urothelium [3,4] as well as from the parasympathetic postganglionic terminals, [8] or from smooth muscle [9]. Like ATP, NO originate from neuronal sources other than the urothelium [10]. To determine the contribution of these two sources to the overall release, we measured ATP and NO release in intact and in urothelium-free preparations. In some species (human, pig) the urothelium can be easily separated from the strips [11-13], making it easy to obtain an urothelium-free preparation. However, in the rat the urothelium is strongly attached to the smooth muscle layer. Its removal is cumbersome, many times incomplete and, in addition, the smooth muscle layer may be damaged.

In isolated blood vessel preparations, it has been demonstrated that gentle wiping on the intimal surface damages the endothelium and leads to profound changes in the contractile function [14]. We wanted to test whether a gentle wiping action on the urothelial side of the bladder would: 1) change the basal and stimulated release of ATP and NO and, 2) change the contractile function of bladder strips.

## Methods

### 2.1. Preparation and treatments

All the experiments were carried out in accordance with the NIH Guide for the Care and Use of Laboratory Animals and were approved by the Institutional Animal Care and Use Committee of Baylor College of Medicine.

Female Sprague-Dawley rats weighing 250-300 grams were used for these experiments. After the bladder was removed from rats anaesthetized with isoflurane, the animals were euthanized in CO_2 _chambers. The bladders were cut in half; one half was left intact while the other half was gently swabbed with a Q-tip in single longitudinal sweeps to remove the urothelium. Two longitudinal strips were prepared from each half and placed into a tissue bath containing oxygenized Krebs solution.

### 2.2. Histological examination of bladder strips

Pieces of intact and swabbed strips were transferred to a Petri-dish containing 4% paraformaldehyde and soaked overnight. The tissues were then embedded in paraffin, and 5 μm slices were cut and stained with hematoxilin-eosin. The slides were examined and photographed under a light microscope with 200 times magnification.

### 2.3. Measurement of bladder contractility

Intact and swabbed strips were mounted in organ baths of 5 mL in Krebs solution containing (mM): NaCl (113.0); KCl (4.7), CaCl2 (1.25), MgSO4 (1.20), NaHCO3 (25.0), KH2PO4 (1.2); d-Glucose (11.5) and bubbled with 95% O_2_/ 5% CO_2 _at 37° C. Trains of square wave pulses (25 V/cm; 0.25 ms, 10 Hz 100 shocks in every 100 s) were delivered by a Grass S88 stimulator (Astromed, RI) via platinum wire electrodes inserted from the top and the bottom of the baths. The isometric contractions were measured with force transducers (F100, WPI) connected to a bridge amplifier (TBM4, WPI, Sarasota, FL), and the contractile data were collected with a computerized data acquisition system. An initial 10 mN pretension was applied at the beginning of the experiments. After a 30 min equilibrium time, we started the stimulation and data collection. Drugs were injected into the bath. The contractile force (mN) was standardized on the basis of the cross-sectional area of the bladder strips expressed as mN/mm^2 ^that was calculated from the strip weight, length, and specific density (1.06 kg/L) as described earlier [15].

### 2.4. Measurement of ATP and NO release

Bladder strips (either intact or with swabbed urothelium) were mounted in a heated tissue bath of 1 mL at 37°C and superfused with oxygenated Krebs at a rate of 1 ml/min. After a 30-minute equilibrium period, one-minute effluents were collected with a fraction collector (Gilson, Middletown MI) for a period of 16 min. The bladder strips were electrically stimulated (50 V, 10 Hz) starting from the 3^rd ^minute for a period of one minute then pharmacologically stimulated with 50 μM carbachol or substance P (SP) for one minute started from the 11^th ^minute of the perfusion. The collected samples were stored in -80°C until the ATP and NO assays were done.

The released amount of ATP and its metabolic derivatives were assayed with HPLC connected to a fluorescent detector (Dynamax, Rainin). The release was expressed as total purine release (i.e. sum of the measured ATP, ADP, AMP, and adenosine release) based on the assumption that all of the metabolites derive from ATP [14]. An aliquot (500 μL) of the effluents was derivatized with chloro-acetaldehyde at 90°C to transform the purines to fluorescent 6-eteno-derivatives using the method described by Todorov et al. [16]. After the precolumn derivatization, 200 μL aliquot of the effluents was injected into a Nova- Pak, Phenyl, 8 × 10 HPLC column (Waters, Milford, MA). The fluorescent detector was set at 230 μm excitation and 420 μm emission wavelengths. The separation of purines was performed with a two-pump gradient system (Gilson 306) with a pump speed of 2 mL/min applying 50 μM phosphate buffer (Buffer A) and 25% methanol phosphate buffer (Buffer B). A linear gradient was run between 3 and 15 minutes from 0% - 100% of Buffer B. Both the pump control and the peak integration were carried out with HPLC software (Unipoint, Gilson, Midtown, WI). The peak areas of the samples were compared with those of standards (ATP, ADP, AMP and adenosine). The quantity of released purines was expressed on the basis of the strip weight in pmol/mg.

NO release was measured with a nitric oxide analyzer (NOAH; GE) by reducing the nitrate and nitrite to NO in the effluents injected into the reaction chamber with vanadium chloride at 95°C. At the beginning of every assay a nitrate standard curve was constructed. Then, 20 μL effluents were injected into the reaction chamber, and the area of the obtained peak was compared to that of the standard curve. The corresponding release values of NO were normalized by the weight of the bladder strip.

### 2.5. Statistical analysis

For statistical analyses one way ANOVA followed by multiple comparisons post-test (Dunnett test) was used. Data were presented as mean ± S.E.; P < 0.05 was considered statistically significant. Statistical analysis and graphics were performed with Prism 4 (Graph Pad Software, San Diego CA) computer program.

### 2.6. Drugs

Alpha-beta methylene ATP (α, β meATP), carbachol, atropine, Substance P, vanadium chloride, ATP, ADP, AMP, adenosine, sodium nitrate, as well as all constituents of Krebs solution, were purchased from Sigma-Aldrich (St Louis, MO).

## Results

### 3.1. Change in bladder morphology after swabbing of the mucosal side

**Figure 1:**
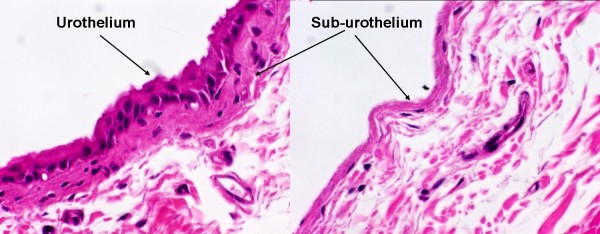
**Representative microscopic picture of the intact (left panel) and swabbed (right panel) bladder strips**. The slides were stained with H & E. The micrographs were taken with 200 × magnification. Note that in the right panel the urothelium is missing while the suburothelial layer is preserved.

In Figure 1 the micrograph clearly shows the presence of the urothelial layer in the intact strip (left panel) while the absence of the epithelium is apparent in the swabbed strip (right panel) while the suburothelial layer is preserved.

### 3.2. Change in bladder contractility

**Figure 2:**
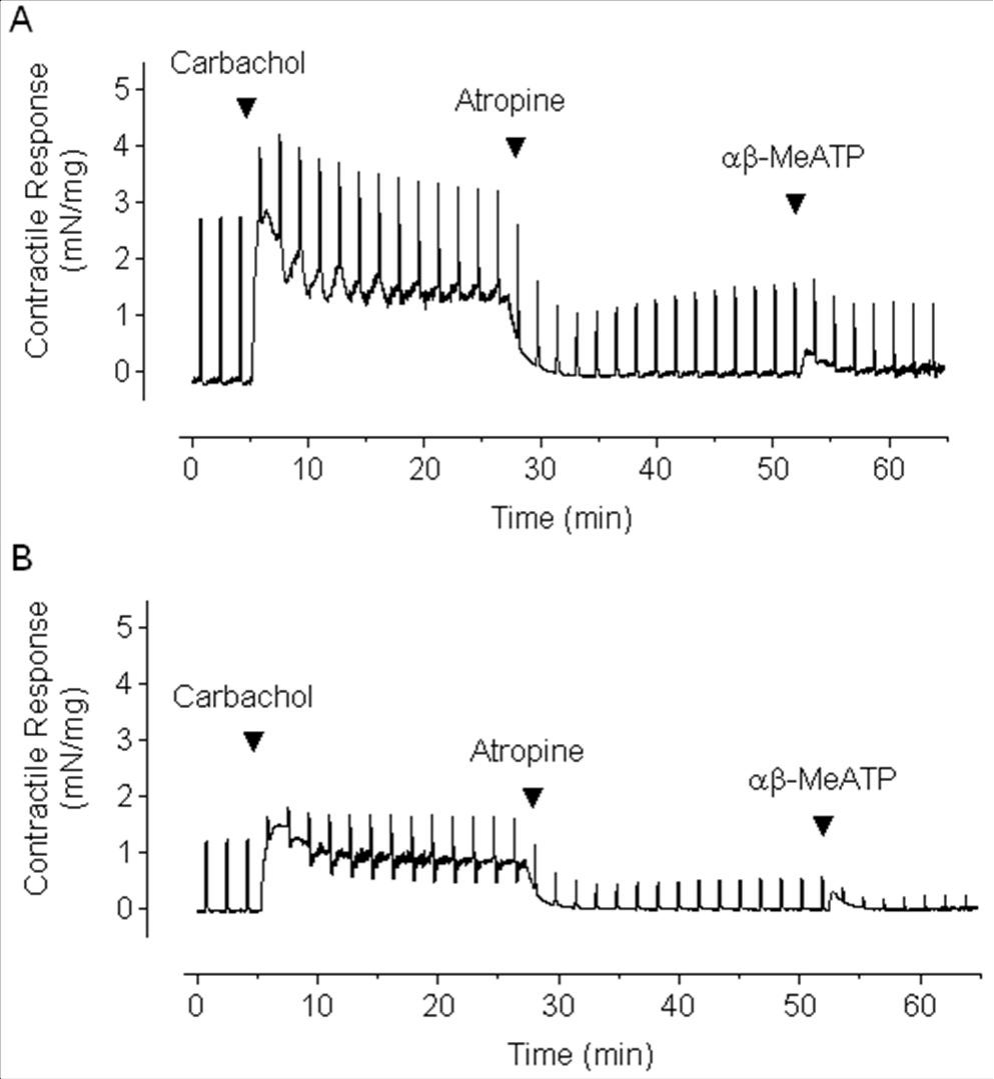
**Representative tracing of contractile response of intact (A) and swabbed (B) bladder strips**. The strips were electrically stimulated and carbachol 50 μM, atropine 1 μM, and α, β meATP (50 μM) were sequentially added into the organ bath. Note that the amplitude of the contractions was smaller in the swabbed preparation.

**Figure 3:**
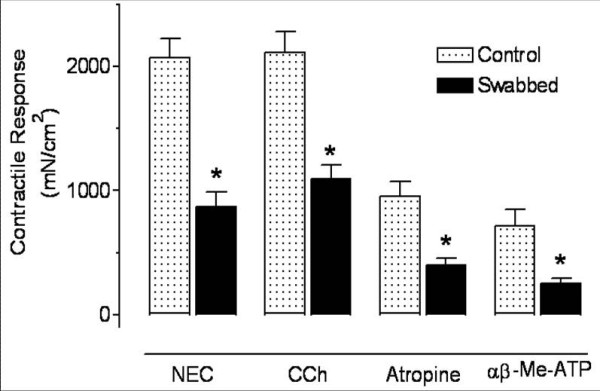
**Contractile response of intact and swabbed bladder**. The contractile force was standardized on the basis of the cross-sectional area (mN/cm^2^) calculated from the strip weight and length. Every bladder was cut in half; one half was left intact and the other was swabbed. Note that there was significant difference between the contractile force in intact and swabbed preparations evoked by electrical or chemical stimulation (NEC = Neurally evoked contraction; *P < 0.05; n = 7 per group; paired t-test).

Bladder contractions were evoked by trains of electrical field stimulation (20 Hz for 10 s at 100s intervals) applied to both intact and swabbed strips. During electrically evoked contractions, carbachol (50 μM) was added to the tissue bath. The application of carbachol increased the tone of the bladder strips on which the electrically evoked contractions were superimposed. Subsequently, 1 μM atropine was added to the bath and quickly reduced the elevated basal tone to the baseline level (Figure 2). Next, α, β meATP (50 μM) was injected into the bath, and that elicited contractions by activating the smooth muscle P2X1 receptors (Figure 2 and 3). As shown in Figure 3, the urothelium-free preparations produced contractions of significantly lower amplitude in response to both electrical and pharmacologic stimulation. In a separate set of experiments, atropine (1 μM) was applied alone to the electrically stimulated intact and swabbed strips to unmask the non-adrenergic, non-cholinergic (NANC) part of the contractions. The neurally evoked contractions were inhibited by atropine to the same extent in both normal and swabbed bladder strips (55% and 49.5%, respectively p > 0.05; n = 6) (data not shown).

### 3.3. Change in evoked release of ATP and NO

#### 3.3.1. Effect of electrical stimulation

**Figure 4:**
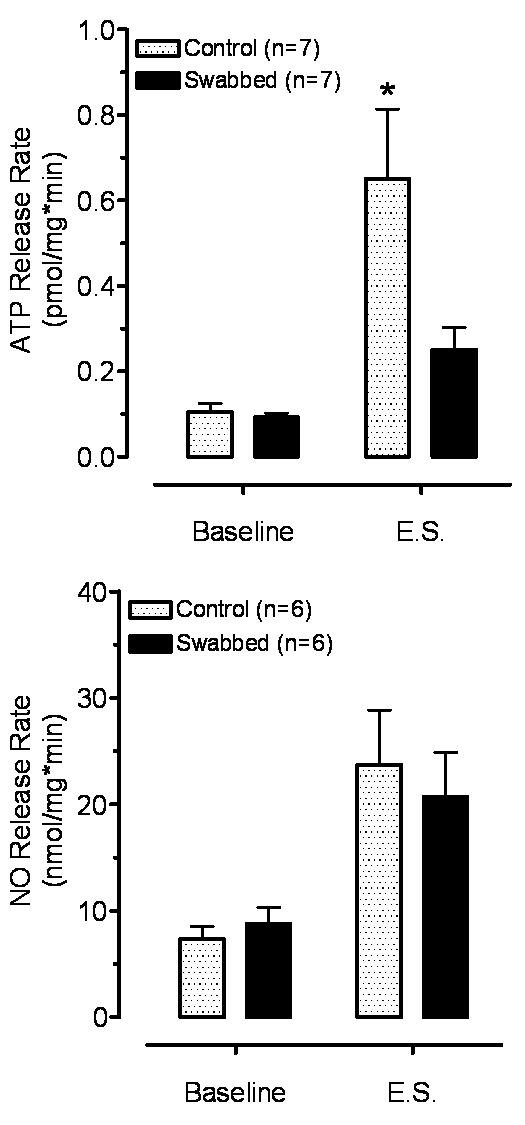
**Effect of electrical stimulation (10 Hz, 1 min, 600 shocks) on ATP (A) and NO (B) release in intact and swabbed bladder strips**. Both ATP and NO release was significantly increased over the baseline in Intact strips (*P < 0.05). In swabbed preparations the ATP release was not significantly different from baseline (P > 0.05), while NO release was still significantly elevated (*P < 0.05). The release of NO was not affected by swabbing (P > 0.2)

The strips were electrically stimulated in the 3^rd ^minute of the perfusion for 1 minute, with application of 1200 shocks at a frequency of 20 Hz. The electrical stimulation significantly increased ATP release over the basal release in the intact but not in the swabbed preparations. In addition, the evoked release of ATP in the swabbed preparations was significantly lower than ATP release in intact bladders (Figure 4A).

In contrast, electrical stimulation increased NO release significantly beyond that of basal release in both intact and swabbed preparations. The evoked release of NO from swabbed strips was not significantly different from intact strips (Figure 4B).

#### 3.3.2. Effect of Carbachol

**Figure 5:**
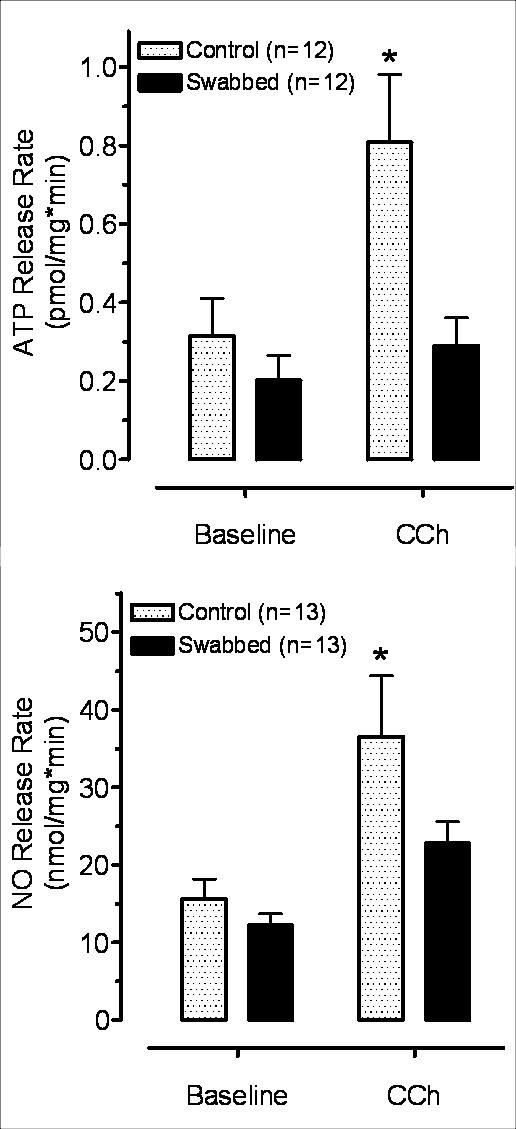
**Effect of carbachol (50 μM) on ATP (A) and NO (B) release in intact and swabbed bladder strips**. Carbachol significantly increased ATP release over the baseline in intact (*P < 0.05) but not in swabbed preparations (P > 0.05). Similarly NO release was significantly higher in intact preparations but did not change in swabbed strips (P > 0.05).

After pharmacological stimulation was applied with the cholinergic agonist carbachol (50 μM), ATP release was significantly increased from the intact but not from the swabbed preparations (Figure 5A).

Carbachol also induced a statistically significant increase in NO release in intact preparations. However, in swabbed strips, it did not increase NO release significantly above the baseline (Figure 5B).

#### 3.3.3. Effect of substance P

**Figure 6:**
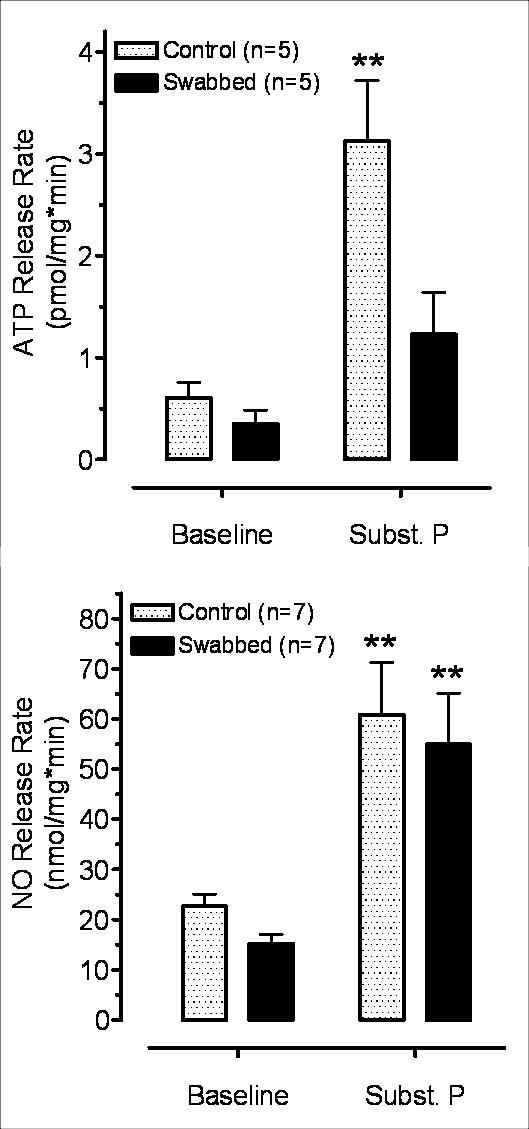
**Effect of substance P (5 μM) on ATP (A) and NO (B) release in intact and swabbed bladder strips**. Substance P significantly increased ATP release (**P < 0.01) over the baseline in intact but not in swabbed preparations (P > 0.05). NO release was significantly increased both in intact and swabbed preparations to the same extent (**P < 0.01).

The neurokinin agonist substance P significantly increased ATP release in intact preparations as compared to the basal release, but it failed to increase ATP release in swabbed bladder strips. The evoked ATP release was significantly lower in swabbed preparations than in intact ones (Figure 6A).

The NO release was significantly enhanced by substance P in both intact and swabbed preparations. Essentially, the evoked NO release was almost identical in both kinds of preparations. Thus, like in electrical stimulation, swabbing did not affect NO release induced by substance P (Figure 6B).

### 3.4. Basal release of ATP and NO

There was no significant difference (P > 0.05) between the basal release of either ATP or NO in intact and swabbed bladder strips (Figures 4, 5, 6).

## Discussion

We investigated the effect on bladder contractile response and on the release of ATP and NO of using a gentle rubbing motion (swabbing) on the urothelial surface. The underlying principles of this methodology are somewhat similar to those described by Furchgott and Zavadski [17] for arterial preparations; namely, that 1) the internal lining of the rabbit aorta was very sensitive to mechanical handling, such as wiping, and that 2) the endothelium-free preparation would respond with a significant change in contractile function and a dramatic decrease of NO production [14,17].

We used cotton swabs to perform a wiping action on the urothelial surface of the bladder. The microscopic pictures indicate that the urothelium is absent, and the absence of urothelium coincided with a significant deterioration in contractile function of the bladder. The amplitude of the contractions evoked by 1) electrical stimulation, 2) injection of α, β meATP, or 3) addition of carbachol to the organ bath was diminished in swabbed bladder strips. Our results differ from those of other investigators who unequivocally demonstrated that the contractile force in urothelium-free bladder preparations increased in various species, such as human [11], pig [12], and guinea pig [18]. Their results suggest that an inhibitory factor derived from the urothelium is tonically inhibiting bladder contractions. This discrepancy may be explained by the difference between our technique of removing the urothelium by swabbing and their technique of surgical removal [11,12]. It is possible that the surgical method removes both the urothelial and the suburothelial layers, whereas a gentle swabbing motion on the urothelial surface may spare the deeper suburothelial layers. Consequently, the source of the urothelium-derived inhibitory factor may be found within the suburothelium rather than the urothelium (Table 1). An alternative possibility is that species differences may account for altered functional role of the urothelium-derived inhibitory factor in the rat bladder.

**Table 1:** The difference between the surgical and mechanical (swabbing) removal of urothelium on ATP release and contractile response of the bladder.

Procedure	Contractility	Basal release (ATP)	Evoked release (ATP)
**Surgical removal [Reference #]**	Increase [11,12]	Decrease [13]	Complete inhibition [13]

**Swabbing**	Decrease	No change	Complete inhibition

Some data were taken from papers indicated by the reference numbers.

Our results are consistent with previous findings that cryo-injury to the serosal surface of the bladder *in vivo *reduces the amplitude of contractions of electrically or pharmacologically stimulated bladder strips [15]. It seems that swabbing the mucosal layer causes an acute injury that results in a diminished contractile response. In cryo-injured preparations, the decrease in contractile amplitude after electrical stimulation coincided with a diminished NANC response because atropine exerted a higher inhibitory action in the injured preparations than in the intact ones. However, in the present experiments the NANC response did not change (i.e. atropine effects did not change with swabbing).

Although both NO and ATP can be released from urothelium [1,3,4] and from nerve terminals in the smooth muscle layer [10], mounting evidence supports the fact that most of the release occurs from the urothelium [7,19]. Interestingly, the basal release of NO and ATP from the intact strip was not significantly different from that from the swabbed strips, suggesting that the non-evoked release of both transmitters may not derive from the urothelium.

The evoked releases of ATP and NO from the intact and swabbed strips show different patterns. We found, as did other investigators, that electrical stimulation increased ATP release from normal strips [13]. However, since the electrically evoked release of ATP is not significantly different from the basal release in swabbed preparations, we conclude that an intact urothelium is a prerequisite for evoking an increase in ATP from bladder strips. On the other hand, NO release was equally robust in both intact and swabbed strips after electrical stimulation suggesting that NO release derives predominantly from deeper suburothelial and/or smooth muscle layers.

When the cholinergic receptors were activated in intact bladder strips by carbachol, the release of both ATP and NO was greatly enhanced. In the swabbed preparations; however, both ATP and NO release were diminished, indicating that the cholinergic receptor that mediates the release of these transmitters is localized in the urothelium. The fact that no significant amount of ATP release was measured from the smooth muscle layer in the absence of urothelium is in contradiction with results described with other smooth muscle preparations (i.e. ileum) where carbachol evoked significant amounts of ATP release [9]. Moreover, in our lab we could evoke ATP release with carbachol in primary smooth muscle cell culture [Unpublished observations]. Thus, it is possible that the urothelium may have a regulatory role in the release of ATP for cholinergic-evoked ATP release in bladder strip preparation. This is consistent with the findings of others that the muscarinic agonist, oxotremorine induced release of ATP from cultured rat urothelial cells [20]. In addition, other investigators have provided indirect evidence that carbachol enhances the release of NO in vivo in rat bladder preparations [21]

It is known that substance P is a neuromodulatory peptide that can be released from bladder afferent terminals. However, the receptors that it activates are more widely distributed and they are expressed on smooth muscle as well as on afferent terminals [22,23]. Substance P significantly increased both ATP and NO release from intact strips. However, in swabbed preparations, ATP release evoked by substance P was significantly less than that in intact bladder strips while the evoked NO release was similar. Thus, the substance P-evoked release of ATP, similar to the response demonstrated with carbachol, is urothelium-dependent (i.e. ATP is released after urothelial neurokinin receptors have been activated). On the other hand, NO release induced by substance P is localized in the suburothelial and/or smooth muscle layers because NO release was not influenced by urothelium removal.

## Conclusions

We suggest the following: 1) The urothelium can be selectively removed by a gentle swabbing motion that leaves the deeper suburothelial layers intact; 2) Removal of the urothelial layers leads to diminished bladder contractions in contrast to the observations of other investigators who found an increased contractile response; 3) Intact urothelium is a prerequisite for evoked release of ATP; 4) NO release secondary to electrical or peptidergic stimulation is independent of the intact urothelium, and 5) Cholinergic-activated NO release is significantly augmented by an intact urothelium.

## Competing interests

The authors declare that they have no competing interests.

## Authors' contributions

AM and DAG contributed equally to this manuscript; they performed the contractile experiments, the stimulation protocols for transmitter release and the assessment of ATP and nitric oxide release. They also participated in analysis and figure generation. CPS and TBB participated in the design and planning of experiments, interpretation of results and writing of the manuscript. GTS is the principal investigator of the study; the idea of swabbing the urinary bladder was his, he wrote the manuscript and supervised the design, planning, analysis, figure generation, interpretation and writing of the manuscript. All authors read and approved the final version of the manuscript.

## Pre-publication history

The pre-publication history for this paper can be accessed here:

http://www.biomedcentral.com/1471-2490/10/10/prepub
